# Influence of age and sex on microRNA response and recovery in the hippocampus following sepsis

**DOI:** 10.18632/aging.203868

**Published:** 2022-01-30

**Authors:** Asha Rani, Jolie Barter, Ashok Kumar, Julie A. Stortz, McKenzie Hollen, Dina Nacionales, Lyle L. Moldawer, Philip A. Efron, Thomas C. Foster

**Affiliations:** 1 Department of Neuroscience, McKnight Brain Institute, University of Florida, Gainesville, FL 32611, USA; 2 Department of Surgery, University of Florida, Gainesville, FL 32611, USA; 3 Genetics and Genomics Program, University of Florida, Gainesville, FL 32611, USA

**Keywords:** aging, sepsis, hippocampus, microRNA, sex dimorphism

## Abstract

Sepsis, defined as a dysregulated host immune response to infection, is a common and dangerous clinical syndrome. The excessive host inflammatory response can induce immediate and persistent cognitive decline, which can be worse in older individuals. Sex-specific differences in the outcome of infectious diseases and sepsis appear to favor females. We employed a murine model to examine the influence of age and sex on the brain's microRNA (miR) response following sepsis. Young and old mice of both sexes underwent cecal ligation and puncture (CLP) with daily restraint stress. Expression of hippocampal miR was examined in age- and sex-matched controls at 1 and 4 days post-CLP. Few miR were modified in a similar manner across age or sex and these few miR were generally associated with neuroprotection against inflammation. Similar to previous work examining transcription, young females exhibited a better recovery of the miR profile from day 1 to day 4, relative to young males and old females. For young males and all female groups, the initial response mainly involved a decrease in miR expression. In contrast, old males exhibited only upregulated miR on day 1 and day 4 and many of the miR upregulated on day 1 and day 4 were linked to neurodegeneration, increased neuroinflammation, and cognitive impairment. The results emphasize age and sex differences in epigenetic mechanisms that likely contribute to susceptibility or resilience to cognitive impairment due to sepsis.

## INTRODUCTION

Sepsis is defined as life-threatening organ dysfunction caused by a dysregulated host immune response to infection [1–4]. Sepsis is surprisingly common with nearly two million hospital admissions annually in the United States, [2, 3, 5]. Furthermore, sepsis can induce immediate and persistent cognitive decline, with worse outcomes in older and Alzheimer’s Disease and Alzheimer’s Disease Related Dementia (AD/ADRD)-diagnosed patients [6]. Of note, sepsis-induced neurocognitive pathology in patients without AD/ADRD has been labeled sepsis-associated encephalopathy and is commonly seen in older adults [7]. In fact, sepsis has been labeled ‘*a disease of the aged*,’ as 60% of septic individuals are older than 65 years [8, 9]. Greater than 50% of sepsis survivors suffer from cognitive dysfunction after hospital discharge, including issues with general memory, attention, verbal fluency, and executive function [10]. Also, a recent nationwide population-based study revealed that dementia is commonly present in a substantial proportion (>11%) of adults ≥65 years of age hospitalized with sepsis [11].

The response to sepsis and recovery from sepsis is influenced by age and sex. Examination of the molecular and physiological response to systemic inflammation in males indicates that diminished cogitation involves impaired synaptic function in the hippocampus, including microglial-mediated synapse elimination, early after a “cytokine storm” [12, 13]. Following this initial response, young animals exhibit resilience and recovery of cognitive and synaptic markers, which may be absent with advanced age [13–15]. Furthermore, there is a sexual dimorphism in response to sepsis with females exhibiting better outcomes [16, 17]. Our recent work examining the hippocampal transcriptome in age and sex-matched controls (i.e., no sepsis day 0) and at 1 and 4 days post-cecal ligation and puncture (CLP) confirmed age and sex differences in gene expression [15]. In general, females were better able to resolve sepsis induced gene changes. In addition, older male mice exhibited a delayed and prolonged response to sepsis. Hypothesized mechanisms for age and sex related differences in the brain’s response to sepsis include epigenetic regulation, with most of the work focused on DNA methylation [18–24].

Epigenetic regulation can also occur through microRNAs (miRs), a family of non-coding small RNAs that can post-transcriptionally regulate protein expression by inhibiting mRNA translation or promoting mRNA degradation [25]. Expression of miRs provides biomarkers of cellular senescence, aging phenotypes, and disease [26–29]. Indeed, many of the miRs involved in immune regulation are altered during aging [30]. While sexually dimorphic differences in brain miR have been described for development and stroke [31], little is known about the role of miR in mediating age and sex differences in the response to sepsis. To examine the role of miR in mediating age and sex differences, we characterized the expression of miR in the hippocampus on day 1 and day 4 after sepsis in young and aged male and female mice. In many cases, the tissue was from the same mice used to describe gene expression [15]. The miR expression results are consistent with mRNA expression in that females, particularly young females, resolve quickly and older males exhibit a prolonged response. Examination of the relationship between increased miR expression and mRNA in older males indicated little predictability for individual miR and mRNA on day 1, likely due to changes in a number of factors associated with fluctuating cytokines. In contrast, a decrease in mRNA expression was more likely on day 4 and linked to multiple miR, directed against specific mRNA. The results emphasize age and sex differences in examining the markers and mechanisms of the response and recovery from sepsis, as well as the need for precision/personalized therapeutics to address post-septic cognitive decline.

## MATERIALS AND METHODS

### Animals

**Figure 1:**
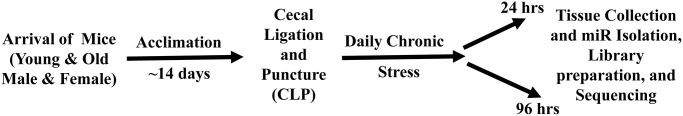
**Schematic diagram of the experimental paradigm for sepsis induction, daily chronic stress (DCS), and tissue collection.** Young adult (~4 months) and old (~20 months) male and female mice were purchase from Jackson Laboratory (JAX Bar Harbor, ME). Prior to initiation of the experiment, mice were acclimated to a 12-hour light-dark cycle for a minimum of 14 days. Sepsis was induced by employing cecal ligation and puncture (CLP) under isoflurane anesthesia. DCS was conducted by placing mice in weighted plexiglass animal restraint holders (Kent Scientific; Torrington, CT) for 2 hours daily commencing the day after CLP. Mice were euthanized for tissue collection either 24 or 96 hours post CLP+DCS. The hippocampus was dissected, flash frozen, and stored at −80, for miR isolation and sequencing.

A schematic of the experimental paradigm is provided in Figure 1. All animal experiments were approved by the University of Florida Institutional Animal Care and Use Committee and followed Animal Research Reporting of *In Vivo* Experiments (ARRIVE) guidelines (https://www.nc3rs.org.uk/arrive-guidelines). The animals were cared for and used according to the Guide for the Care and Use of Laboratory Animals [32]. Young (~4 months) and old (~20 months) adult C57BL/6J (B6) mice of both sexes (young male = 12; old male = 12; young female = 12; old female = 12) were purchased from Jackson Laboratory (JAX; Bar Harbor, ME). Mice were cared for by the University of Florida Animal Care Services and housed in transparent cages (three to four animals of same sex/age per cage) under specific pathogen-free conditions in a single room. Animals were provided standard irradiated pelleted diet and water ad libitum for the duration of the study. Prior to initiation of the experiment, mice were acclimated to a 12-hour light-dark cycle for a minimum of 14 days. Due to the coprophagic nature of mice, this assured that mice in the same cage would have similar microbiota composition and structure [33]. Only animals of the same sex, age, and treatment group were housed together.

### Intra-abdominal sepsis and daily chronic stress model

In order to recapitulate the human condition of abdominal sepsis, a murine model of sepsis and persistent inflammation, previously described by our laboratory, was utilized [34]. Briefly, general anesthesia was induced with inhaled isoflurane and CLP was performed via a midline laparotomy with exteriorization of the cecum to induce a model of sepsis. The cecum was ligated with 2-0 silk suture 1 cm from its tip, a 25-gauge needle was used to puncture the cecum, and the laparotomy was closed in one layer with surgical clips. Buprenorphine analgesia was provided for 48 hours post-surgery. Imipenem monohydrate (25 mg/kg in 1 mL 0.9% normal saline) was administered subcutaneously 2 hours post-CLP and then continued twice daily for 72 hours. Subsequently, we added a component of daily chronic stress (DCS). DCS was conducted by placing mice in weighted plexiglass animal restraint holders (Kent Scientific; Torrington, CT) for 2 hours daily commencing the day after CLP. DCS was combined with CLP (CLP+DCS) to mimic the stress that occurs in patients when residing in an intensive care unit and better reflects human sepsis relative to CLP alone [34, 35]. This model was previously used to describe age and sex-related changes in hippocampal transcription [15]. The CLP+DCS mice, along with mixed-sex naïve mice (no CLP, no DCS, no antibiotics, and no fluid resuscitation) were euthanized on day 1 or 4 post-CLP+DCS. Mice were anesthetized with isoflurane (Halocarbon Laboratories, River Edge, NJ) and swiftly decapitated.

The work consisted of young and old female (50%) and male (50%) mice. Of note, the last restraint stress occurred 1 hour prior to sacrifice, and mice that were sacrificed 24 hours after CLP received restraint cone stress 1 hour prior to sacrifice.

### Tissue collection

Animals were sacrificed either 24 hours or 4 days following CLP. Age and sex-matched control groups were sacrificed directly from the home cage, without receiving surgery or anesthesia. Mice were anesthetized with isoflurane (Halocarbon Laboratories, River Edge, NJ) and swiftly decapitated. The brains were rapidly removed and the hippocampi were dissected. All brain samples were flash frozen in liquid nitrogen and were stored at −80°C. One whole hippocampus was used for microRNA sequencing. In some cases, the other hippocampus was used for mRNA sequencing and the results have previously been reported [15].

### RNA isolation

Hippocampus tissue miR was isolated using mirVana miR Isolation Kit (ThermoFisher Scientific, Cat# AM1560) according to the manufacturer’s instructions. The quantity and quality of the RNA was determined by University of Florida Interdisciplinary Center for Biotechnology Research using the Agilent RNA 6000 Pico Kit to determine the concentration of total RNA, and a Small RNA Kit was used to measure the concentration of tissue micro RNA (miR) on the Agilent Bioanalyzer instrument (Agilent Technologies).

### Small RNA library preparation and sequencing

To perform miR profiles, sequencing libraries were prepared using 48 samples (old and young adult males and females) of 12 different groups. For males, examination of miR expression was performed for the same mice in which the mRNA response was examined and included young male control (*n* = 4/4, miR/mRNA), young male 24 hours post-sepsis (*n* = 4/4), young male 4 days post-sepsis (*n* = 4/4), old male control (*n* = 4/4), old male 24 hours post-sepsis (*n* = 4/4), old male 4 days post-sepsis (*n* = 4/4). For females, most of the miR samples were from the same mice in which mRNA expression was measured, particularly for older females. However, due to loss of tissue, some younger females were replaced: young female control (*n* = 4/2, miR/mRNA), young female 24 hours post-sepsis (*n* = 4/1), young female 4 days post-sepsis (*n* = 4/3), old female control (*n* = 4/4), old female 24 hours post-sepsis (*n* = 4/4), and old female 4 days post-sepsis (*n* = 4/3). Methods for miR library preparation and sequencing have previously been published [28, 36, 37]. Briefly small RNA libraries were prepared using the Ion Total RNA-Seq Kit v2 (Thermo Fisher, catalog number 4475936). Each library was barcoded with Ion Xpress RNA Seq-Barcode 01-16 Kit (ThermoFisher, Cat# 4475485) to enable multiplex sequencing. The concentration of the libraries was quantified by the Qubit dsDNA HS Assay (Thermo Fisher, Cat# Q32851). In addition, the size distribution and molar concentration was determined with the High Sensitivity D1000 Screen Tape Kit (5067-5584) on 2200 TapeStation system (Agilent Technologies, Cat#G2964A) according to the manufacture’s protocol.

### Data acquisition, bioinformatics and statistical analysis

Data acquisition and analysis for miR expression was performed as previously described [28, 36, 37]. In brief, on Partek Flow server FASTQ files were trimmed and aligned to the mouse (mm10) genome using Bowtie (version 1.0.0). Normalization was performed on total counts in Partek and genes with an average total count of less than 5 were removed, consistent with our previously published work [28, 36–39]. Statistical filtering was performed using a *p*-value set at *p* < 0.05. Features list that passed the statistical filter were then separated into upregulated or downregulated based on fold change. The data for this study has been uploaded to the Gene Expression Omnibus under the accession number GSE188874.

## RESULTS

**Figure 2:**
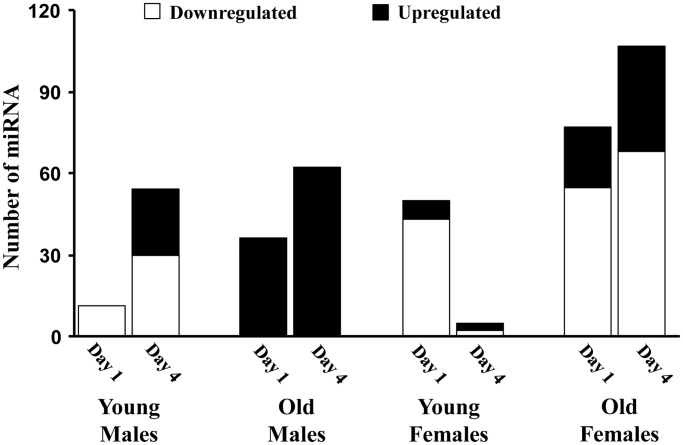
**The number of miRs differentially expressed for each age and sex group.** Summary of the total number of miRs increased (black) or decreased (white) expression in the hippocampus 1 or 4 days after sepsis relative to age-matched controls.

Figure 2 illustrates the global pattern of differentially expressed miRs (DEmiRs) in young and old adult male and female mice, specifically 1 or 4 days following sepsis compared to sex and age-matched controls. In general, females and older animals exhibited a more robust response on day 1, with an increased number of DEmiR. Interestingly, the largest changes were for downregulated miR with the exception on older males, which exhibited only up regulated miR. Young females exhibited considerable recovery on day 4, observed as a marked decrease in the number of DEmiR. For the other groups, there was an increase in DEmiR on day 4, mainly for upregulated miR, except for older females, which exhibited increased expression of up and downregulated miR. The increase in upregulated miR was particularly robust for older males, which exhibited the greatest number of upregulated miR on day 1 and day 4.

### Young adult male mice

On day 1 following sepsis, young males exhibited 11 downregulated miRs compared to controls, in the absence of upregulated miR (Figure 2). On day 4 following sepsis, the number of downregulated miRs increased to 30 and included 2, miR-335-3p and miR-let-7d-3p, which were also decreased on day 1. Expression of miR-let-7d-3p has been linked the regulation of the inflammatory response [40, 41] and expression of miR-335-3p has been linked to the response to stress [42] and regulation of neuroprotection and neurodegeneration [43, 44]. In addition, elevated expression of miR-335-3p has been associated with poorer memory during aging [45] and inhibition of estrogen receptor expression [46]. Upregulation was observed for 24 miRs on day 4.

### Old adult male mice

In contrast to young males, the response in old males was limited to up regulation of miRs for both day 1 and day 4. For older males, 36 miRs were upregulated on day 1 and the number of upregulated miRs increased to 62 on day 4, with 22 increased on both days, relative to controls (Table 1). Three of the miRs upregulated on days 1–4 (miR-223-3p, miR-98-3p, miR-662-5p) are linked to the X-chromosome. Examination of the pattern of expression for these 22 miRs indicated that 19 continued to increase from day 1 to day 4. Although expression was above baseline, 3 miRs exhibited evidence of returning towards baseline. No miRs were affected in the same direction on day 1 in young and older males. There were 6 miRs that exhibited an increase on day 4, in young and old septic males. These miRs have been linked to inhibition of inflammation and neuroprotection (miR-223-3p, miR-544-5p, miR-219a-5p, miR-15a-5p) [47–54] and neural development and cognition (miR-190a-3p, miR-344-3p) [55, 56]. In addition, 5 miRs, which were increased in older males on day 4, exhibited downregulation in young males on day 4. Again, these miRs are associated with neuroprotection and reduced neuroinflammation, including let-7c-5p [57]. However, upregulation of let-7b-5p [58, 59] and let-7a-5p [60] in older males may be markers of metabolic stress consistent with a delayed/prolonged response in older males during a time when younger animals are exhibiting resolution of inflammation.

**Table 1:** Mean ± SEM fold change, relative to the mean of old control males, for normalized counts of 22 miRs that increased expression on day 1 and day 4 of sepsis in old males.

**miR**	**Day 1**	**Day 4**	**Change Day 1 to Day 4**
mmu-miR-381-3p	1.40 ± 0.04	1.46 ± 0.09	up
mmu-miR-872-3p	1.45 ± 0.09	1.63 ± 0.24	up
mmu-let-7f-1-3p	1.51 ± 0.23	1.39 ± 0.12	down
mmu-miR-7a-1-3p	1.52 ± 0.07	1.49 ± 0.17	down
mmu-miR-212-3p	1.52 ± 0.10	1.58 ± 0.23	up
mmu-miR-31-3p	1.60 ± 0.07	1.59 ± 0.19	down
mmu-miR-30b-5p	1.61 ± 0.15	2.10 ± 0.35	up
mmu-miR-342-3p	1.61 ± 0.09	1.76 ± 0.31	up
mmu-miR-323-3p	1.64 ± 0.11	1.86 ± 0.46	up
mmu-miR-223-3p	1.65 ± 0.26	2.50 ± 0.70	up
mmu-miR-672-3p	1.66 ± 0.25	2.17 ± 0.41	up
mmu-miR-15a-5p	1.71 ± 0.13	1.53 ± 0.19	down
mmu-miR-30c-5p	1.72 ± 0.19	1.99 ± 0.18	up
mmu-miR-33-3p	1.72 ± 0.13	1.53 ± 0.12	down
mmu-miR-15b-5p	1.74 ± 0.03	1.87 ± 0.43	up
mmu-miR-98-3p	1.80 ± 0.35	1.65 ± 0.19	down
mmu-miR-106b-5p	1.82 ± 0.15	1.90 ± 0.30	up
mmu-miR-340-5p	1.84 ± 0.16	2.82 ± 0.94	up
mmu-miR-672-5p	1.85 ± 0.37	2.82 ± 0.99	up
mmu-miR-467e-5p	1.96 ± 0.37	1.68 ± 0.36	down
mmu-miR-190a-3p	1.97 ± 0.18	2.05 ± 0.34	up
mmu-miR-362-5p	2.80 ± 0.58	3.88 ± 0.72	up

### Young adult female mice

For young females on day 1 following sepsis, the number of DEmiRs was approximately 5 fold more than young male; however, like males, most of the miRs, 43 out of 50, were downregulated (Figure 2). In addition, while young males exhibited an increased number of DEmiRs on day 4, young females exhibited a reduced number of DEmiRs on day 4 (3 upregulated and 2 downregulated), consistent with increased rate of recovery for young females over this time period [15]. The DEmiRs for young females were not common across days, such that no miRs exhibited a similar directional change on day 1 and day 4. Furthermore, the DEmiRs were not the same as that observed for young males, with no common up or downregulated miRs on day 1 and only two miRNAs (miR-383-5p, miR-1249-3p) were downregulated on day 4 in both young females and young males. Downregulation of miR-383-5p may provide a neuroprotection against inflammation and associated oxidative stress [61]. Thus, for young animals, the initial response on day 1 is mainly a downregulation of miRs; although the specific miRs differ across sex, suggesting a sexually dimorphic response to sepsis. Furthermore, young females exhibited considerable recovery from day 1 to day 4.

### Old adult female mice

Similar to the age comparison for young and old males, older females were more responsive than young females, on day 1 after sepsis, exhibiting 22 upregulated and 55 downregulated miRs (Figure 2). Three miRs were upregulated in young and older females on day 1 (miR-190a-3p, miR-let-7a-1-3p, miR-3085-3p). Interestingly, miR-190a-3p, which is neuroprotective [62, 63] was also upregulated in old males on day 1 and both young and old males on day 4. miR-let-7a is induced by systemic inflammation and regulated by estradiol [64]. Seven miRs were downregulated on day 1 in both young and old females (miR-127-3p, miR-222-3p, miR-299a-3p, miR-221-3p, miR-337-5p, miR-541-5p, miR-652-3p). Three are linked to the X-chromosome (miR-222-3p, miR-221-3p, miR-652-3p). In this case, downregulation has been linked to reduced inflammation (miR-222-3p, miR-221-3p) [65–69]. Thus, on day 1 following sepsis, the expression of miR in young and old females is suggestive of process for neuroprotection and reduced inflammation. No miRs were common for young and old females on day 4, likely due to the considerable recovery of young females.

For both day 1 and day 4 for older females, 18 miRs were downregulated (Table 2) and 3 miRs were upregulated (miR-669b-5p, miR-5121, miR-542-3p). For the downregulated miRs, nine continued to decrease on day 4, while 9 exhibited a return towards baseline. When comparing older males and older females, older females exhibit mainly downregulation of miR on day 1 and day 4, and older males exhibited only upregulated miR. Across the two groups, five miRs (miR-190a-3p, let-7a1-3p, miR-362-5p, miR-31-3p, miR-7b-3p) were up regulated on day 1 in older males and older females. Five different miRs (miR-30c-5p, miR-30b-5p, miR-143-3p, miR-384-5p, miR-380-3p) were up regulated on day 4 across all older animals.

**Table 2:** Mean ± SEM fold change, relative to the mean of old control females, for normalized counts of 18 miRs that decreased expression on day 1 and day 4 of sepsis in old females.

**miR**	**Day 1**	**Day 4**	**Change Day 1 to Day 4**
mmu-miR-320-3p	0.44 ± 0.05	0.71 ± 0.10	up
mmu-miR-383-5p	0.53 ± 0.05	0.65 ± 0.12	up
mmu-miR-135b-5p	0.54 ± 0.06	0.53 ± 0.01	down
mmu-miR-495-5p	0.57 ± 0.01	0.57 ± 0.11	down
mmu-miR-409-5p	0.60 ± 0.06	0.57 ± 0.04	down
mmu-miR-129-2-3p	0.63 ± 0.06	0.55 ± 0.02	down
mmu-miR-322-5p	0.63 ± 0.06	0.35 ± 0.02	down
mmu-miR-377-5p	0.64 ± 0.04	0.50 ± 0.09	down
mmu-miR-671-5p	0.65 ± 0.10	0.72 ± 0.16	up
mmu-miR-370-3p	0.66 ± 0.05	0.65 ± 0.10	down
mmu-miR-324-5p	0.68 ± 0.05	0.72 ± 0.11	up
mmu-miR-125b-1-3p	0.71 ± 0.06	0.73 ± 0.06	up
mmu-miR-135a-5p	0.31 ± 0.03	0.30 ± 0.03	down
mmu-miR-130a-3p	0.39 ± 0.03	0.33 ± 0.08	down
mmu-miR-873a-3p	0.39 ± 0.04	0.41 ± 0.06	up
mmu-miR-26b-5p	0.48 ± 0.01	0.66 ± 0.10	up
mmu-miR-140-5p	0.56 ± 0.04	0.61 ± 0.03	up
mmu-miR-99b-3p	0.60 ± 0.08	0.65 ± 0.07	up

### Relationship between increased miR and mRNA

Theoretically, expression of miR and associated mRNA should be inversely correlated. In this case, we would expect that for the 22 miRs that increased 1–4 days following sepsis in old males, the associated mRNA should exhibit decreased expression. We used mRNA expression previously reported [15] for the same older male animals (*n* = 12), to determine if genes that were significantly (*p* < 0.05) upregulated or downregulated on day 1 and day 4, were associated with the 22 miR that were increased on both days. The predicted mRNA for each of the 22 miRs was obtained using mirWalk database [70].

**Figure 3:**
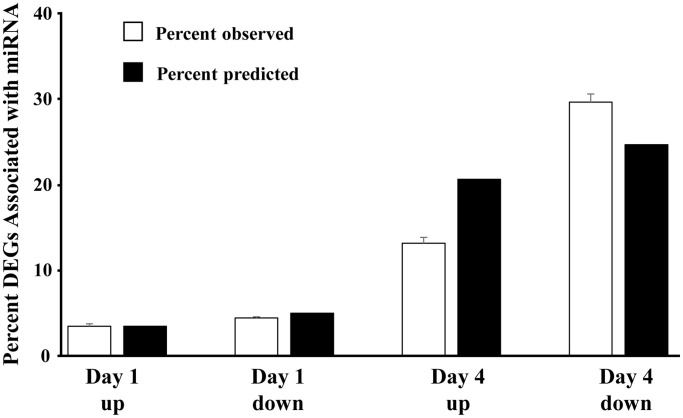
**Direction of mRNA expression associated with increased miR expression on day 1 and day 4 post-sepsis.** The open bars represent the mean + SEM percent of total differentially expressed genes, associated with the 22 miRs that increased in older males on day 1 and day 4, for each category (Day 1 and Day 4 up and downregulated genes). The filled bars represent the percent of differentially expressed genes, relative to day 1 upregulated, which are expected due to an increase in the total number of differentially expressed genes. Note that for day 4, the percent of upregulated differentially expressed genes is 68% of predicted and the percent of downregulated differentially expressed genes is 120% of predicted, suggesting that upregulated miRs are gaining control (i.e., downregulating) the associated mRNA.

Using the previously reported mRNA expression of older males [15], the percent of significantly (*p* < 0.05) differentially expressed genes, linked to each of the 22 miRs within each category (day 1 up 496 genes, day 1 down 701 genes, day 4 up 2932 genes, day 4 down 3508 genes) were determined (number of associated DEGs/total number of significant genes in each category) (Figure 3). The percent of DEGs associated with the miR was greatly increased on day 4. Due to differences in the number of DEGs, we might predict that the proportion linked to the miR would increase from upregulated to downregulated on day 1 (701/496 = 1.41) and on day 4 relative to day 1 upregulated (day 4 up 2932/496 = 5.91; day 4 down 3508/496 = 7.07). However, relative to upregulated genes on day 1, the percent of day 4 upregulated mRNA was 63% of predicted (13.2 observed/20.7 predicted) and the downregulated mRNA was 120% of predicted (29.7 observed/24.7 predicted) (Figure 3). The results indicate that the expected decrease in mRNA expression associated with increased miR expression was more prominent on day 4.

**Figure 4:**
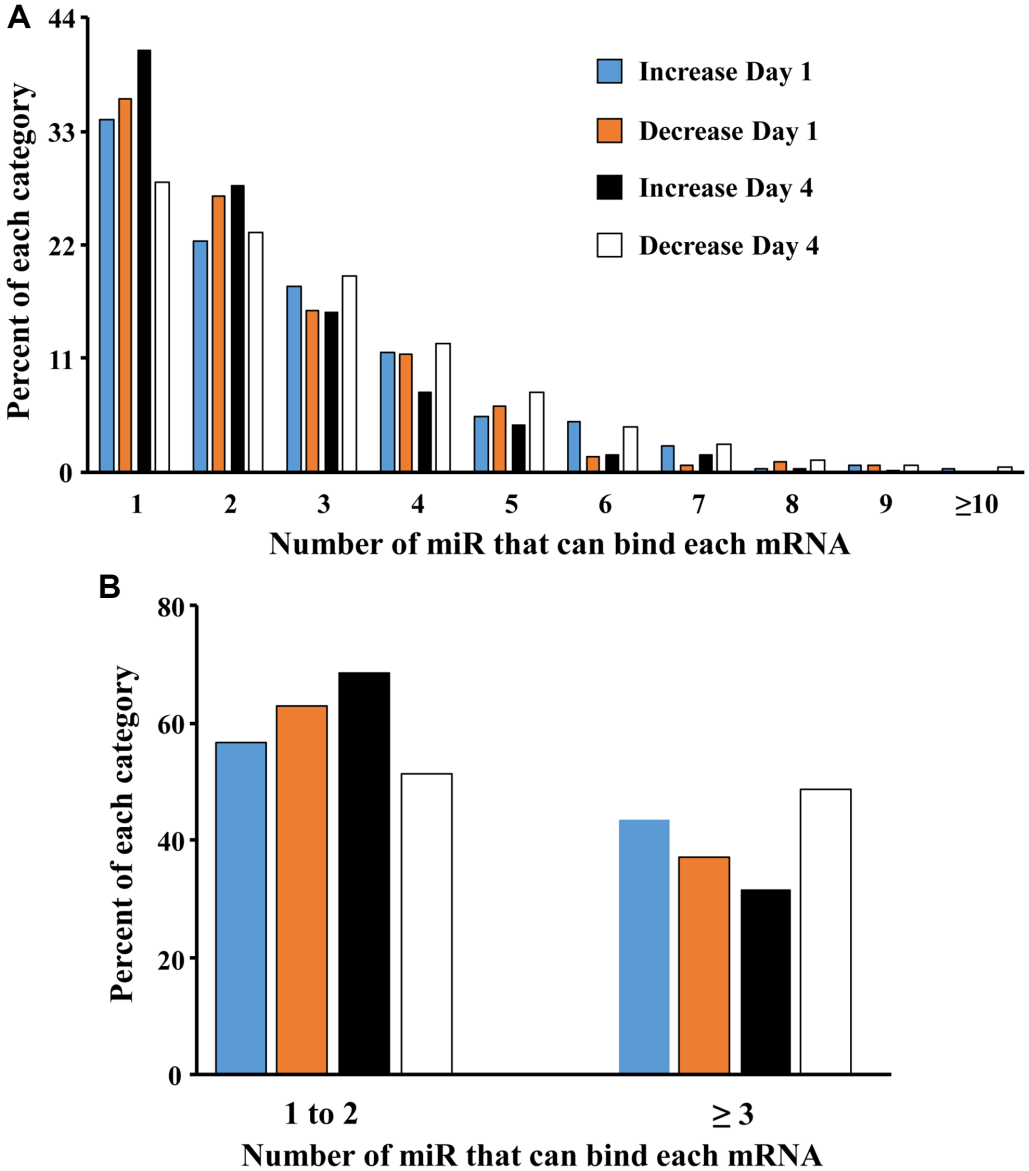
**Increasing the number of miRs associated with an individual gene promotes mRNA downregulation.** (**A**) Each bar represents the proportion of mRNAs (y-axis) that were upregulated on day 1 (blue bar) and day 4 (orange bar) or downregulated on day 1 (black bar) and day 4 (white bar) and were associated with 1 to ≥10 of the 22 miRs (x-axis) that increase in older males. (**B**) Collapsing the data to show that the percent of gene expression associated with 1–2 or ≥3 miRs. Note that on day 4, mRNA associated with ≥3 miRs are more likely to be downregulated.

A mismatch between expression of miR and mRNA is likely due to other transcriptional regulators, including the downregulation of repressor genes that would normally inhibit transcription [71, 72]. The ability of miR to inhibit gene expression maybe enhanced by an increase in the number of different miR that bind the specific mRNA of interest. To examine the effect of increasing number of different miRs that target individual genes, we examined how many of the 22 miR that were upregulated over days 1–4 in older males, targeted each gene that exhibited a significant increase or decrease in expression (Figure 4A). Most (>50%) of the mRNAs were associated with 1–2 miR. However, by day 4, there was a marked increase in the proportion of mRNA that exhibited decreased expression, which were associated with multiple (>2) miRs. Thus, the proportion of decreased mRNA associated with 3 or more miR went from 37% on day 1 to 49% on day 4 (Figure 4B). Together, the results suggest that by day 4, multiple miR appear to gain increasing control over expression of individual mRNA.

## DISCUSSION

Variability in cognitive decline during aging is associated with genetic factors, including sex, and environmental factors, including the history of severe inflammation, that act through epigenetic mechanisms to modify the trajectory of cognitive decline [14, 25, 73]. Sex differences are observed for the response and recovery of brain damage, infectious diseases, and aging [15, 31, 74–76], possibly due to sex steroids, sex chromosomes, and epigenetic differences. Our previous work indicates sex and age differences in the hippocampal transcriptional response and recovery from sepsis [15]. The current study indicates a similar pattern of expression for miR. Over all, females were the most responsive group on day 1. In particular, females exhibited more miR downregulated on day 1 and several were common in young and old females. Several common miRs, which were downregulated on day 1 in young and aged females, are located on the X-chromosome and have been reported to regulate inflammation including miR-222-3p [65, 77], miR-221-3p [69, 78–80], and miR-652-3p [81]. In contrast, older males exhibited increased expression of three different X-chromosome miR (miR-223-3p, miR-98-3p, miR-662-5p). In this case, an increase in miR-223-3p appears to protect against inflammation [47–49]. Anti-inflammatory properties of the sex steroid, estrogen, may contribute to the sexually dimorphic response to inflammation and sepsis and sepsis induced changes in miR may reflect a role for estrogen in recovery from inflammation. For example, young males exhibited downregulation of miR-335-3p on day 1 and day 4, which could enhance expression of estrogen receptor alpha [46]. Similarly, on day 1 young and older females exhibited upregulation of miR-222-3p which could increase estrogen receptor alpha expression [82]. On day 4, young and older males exhibited increased expression of miR-15a-5p, which may regulate estrogen signaling [83]. Indeed, for the 22 miRs that were increased on day 1 and day 4 in older males, several (miR-30b-5p, miR-342-3p, miR-30c-5p, miR-106b-5p, miR-672-5p) have been linked to estrogen responsiveness [84–88].

The marked decrease in miR expression on day 1 may suggest a level of baseline control of gene expression by miR, which is rapidly altered during the cytokine storm. In contrast, the upregulated miR was more prominent on day 4 in males and older females. Relatively few miRs were altered in the same direction across days and were mainly limited to older animals. Again, differences across days may relate to the time course of the cytokine levels (i.e., cytokine storm) and the ability to recover from infection. For older females, 18 miRs were decreased on day 1 and day 4; however, half the miR exhibited evidence of recovery with expression moving towards baseline from day 1 to day 4, consistent with better recovery of females.

Very few miRs were similarly modified across age or sex, particularly for day 1. For some miRs that were consistently downregulated in young males on day 1 and day 4 (miR-let-7d-3p) or downregulated in young males and females on day 4 (miR-383-5p), previous research suggest that downregulation is neuroprotective against inflammation and associated oxidative stress [41, 42, 61]. Similarly, for miRs that exhibited an increase on day 4, in young and old septic males (miR-223-3p, miR-544-5p, miR-219a-5p, miR-15a-5p), the increased expression has been linked to inhibition of inflammation and neuroprotection [47–54]. Three miRs were upregulated in young and older females on day 1 (miR-190a-3p, let-7a-1-3p, miR-3085-3p). Increased expression of let-7a-1-3p [89] is associated with reduced inflammation following spinal cord injury. Increased miR-3085-3p may be responsible for the early inhibition of NFκB signaling [90]. Interestingly, miR-190a-3p, which is neuroprotective [62, 63], exhibited increased expression across sexes, in that it was also upregulated in old males on day 1 and both young and old males on day 4. In contrast, three of the five miRs that were up regulated on day 4 across older male and female animals (miR-143-3p, miR-380-3p, miR-384-5p) may contribute to neurotoxicity [91–96].

Severe systemic inflammation is a negative modifier of the trajectory of cognitive decline [14, 97]. Furthermore, elderly patients are more likely to suffer cognitive impairment after sepsis-associated encephalopathy [6, 7]. Epigenetic changes over the course of aging or due to the history of infection can either prime the brain to respond to immune stimulation or result in immune tolerance [14, 98, 99]. In this way, epigenetics regulates transcriptional responsiveness and susceptibility or resilience to stressors of aging, including systemic inflammation [14, 25, 100, 101]. Table 3 provides a summary of biological functions related to neuroinflammation, neuroprotection, neurodegeneration, and cognition for some of the 22 miRs, which were increased in older male mice, and may contribute to differences in transcription and susceptibility or resilience to cognitive impairment. For example, age-related differences in cognitive impairment, associated with systemic infection, are linked to decreased transcription of hippocampal synaptic genes and an altered transcriptional response to inflammation [13–15]. Thus, the increase in miR-7a-1-3p, miR-33-3p, and miR-362-5p may contribute to decreased expression of neuronal/glial and synaptic genes; while miR-15a-5p, miR-15b-5p, and miR-30c-5p may influence the expression of apoptotic genes observed in older males on day 4 [15]. Nevertheless, for most of the miRs, increased expression is linked to neurodegenerative disease, increased neuroinflammation, and cognitive impairment (Table 3) consistent with enhanced vulnerability of older brains. However, increased expression of miR-223-3p, miR-340-5p, and miR-30b-5p has been linked to anti-inflammatory neuroprotection, suggesting possible resilience mechanisms that may preserve cognition in the face of neuroinflammation [73, 100].

**Table 3:** A summary of biological functions related to neuroinflammation, neuroprotection, neurodegeneration, and cognition for some of the 22 miRs, which were increased in older male mice.

**miR**	**Role in neuroinflammation, neuroprotection, neurodegeneration, and cognition**
miR-106b-5p	Upregulated during neuroinflammation and neurodegenerative disease models [105–109]
miR-15a-5p	Can have pro- or anti-apoptotic activity [53, 54, 110]
miR-15b-5p	Can have pro- or anti-apoptotic activity [110–113]
miR-190a-3p	Biomarker for postoperative cognitive dysfunction [55]
miR-212-3p	Downregulation is a biomarker for neurodegenerative disease [114–116]
miR-223-3p	Inhibition of neuroinflammation [47–49, 117]. Biomarker of sepsis severity [102]
miR-30b-5p	Upregulation is neuroprotective [118–120]
miR-30c-5p	Can have pro- or anti-apoptotic activity [121–123]
miR-31-3p	Role in conditioned place preference [124]
miR-323-3p	Biomarker for cognitive impairment [125, 126]
miR-33-3p	Neurogenesis [127]
miR-340-5p	Anti-inflammatory and neuroprotective [128–130]
miR-342-3p	Upregulated in neuroinflammation and neurodegenerative disease models [131–135]
miR-362-5p	Nervous system development [136]
miR-381-3p	Upregulation during encephalomyelitis [137] and HIV associated with cognitive impairment [138]
miR-7a-1-3p	Promotes generation of oligodendrocytes [139] and regulates excitatory synaptic transmission [140]
let-7f-1	Promotes IL-6 secretion in activated macrophages [141]
miR-98-3p	Upregulated by caloric restriction [142]

One question we attempt to address is whether the increase in miR expression on day 1 and day 4 in older males contributes to the observed change in mRNA expression. Theoretically, expression of miR and associated mRNA should be inversely correlated. For the 22 miRs that increased on day 1 and day 4 in older males, the likelihood that the associated mRNA was increased or decreased was equivalent on day 1. In contrast, an increased propensity for mRNA to exhibit downregulation was evident on day 4. The decrease in mRNA on day 4 may relate to the fact that most of the 22 miRs continued to increase expression from day 1 to day 4. Interestingly, downregulation of mRNA was largely observed for genes, which interact with multiple miRs. The absence of a negative correlation between individual miR and associated mRNA on day 1 may be due other transcriptional regulators activated during or immediately after the cytokine storm associated with sepsis. Previous work suggests that despite an increase in miR, expression of associated mRNA may be increased, rather than decreased, due to ongoing activation of transcription factors or the downregulation of repressor genes, which would normally inhibit transcription [71, 72]. On day 4, upregulated miR in older males appears to gain influence on mRNA expression. In particular, decreased expression is observed for mRNA that can be bound by multiple upregulated miRs.

The molecular mechanisms that underlie age and sex differences in response to inflammation, disease, brain damage, and aging are not fully understood. The results emphasize age and sex differences in epigenetic mechanisms that may contribute to differences in vulnerability to sepsis. Few miR were modified in a similar manner across age or sex; however, these few miR were generally associated with neuroprotection against inflammation. In contrast, older males exhibit increased expression of several miRs linked to neurodegeneration, increased neuroinflammation, and cognitive impairment. The differences may contribute to age and sexually dimorphic responses to sepsis, and emphasize a need for precision/personalized therapeutics to address post-septic cognitive decline. miRs isolated from blood can provide diagnostic and prognostic information concerning sepsis [102, 103] and cognition [26, 28]. Furthermore, systemic delivery of miR may protect against sepsis induced brain injury [104]. Thus, it may be important for future studies to examine the relationship between miRs detected in blood and brain.
